# 4,4′-Bipyridine-1,1′-diium bis­(1,3-benzo­thia­zole-2-thiol­ate)

**DOI:** 10.1107/S1600536812047058

**Published:** 2012-11-24

**Authors:** Yu-Han Jiang, Qi-Ming Qiu, Min Liu, Qiong-Hua Jin, Cun-Lin Zhang

**Affiliations:** a Department of Chemistry, Capital Normal University, Beijing 100048, People’s Republic of China; b The College of Materials Science and Engineering, Beijing University of Technology, Beijing 100022, People’s Republic of China; c Key Laboratory of Terahertz Optoelectronics, Ministry of Education, Department of Physics, Capital Normal University, Beijing 100048, People’s Republic of China

## Abstract

In the title salt, C_10_H_10_N_2_^2+^·2C_7_H_4_NS_2_^−^, the complete 4,4′-bipyridine-1,1′-diium dication is generated by a center of symmetry. In the crystal, N—H⋯N hydrogen bonds are observed between the cations and anions.

## Related literature

For ligands based on 2-mercaptobenzothia­zole in coordination chemistry, see: Chen *et al.* (2010 ▶) and for ligands based on 4,4′- bipyridine, see: Biradha *et al.* (1999 ▶); Ren *et al.* (2004 ▶); Tao *et al.* (2000 ▶); Tong *et al.* (2000 ▶); Xu *et al.* (2012 ▶). For a related structure, see: Deng *et al.* (2005 ▶).
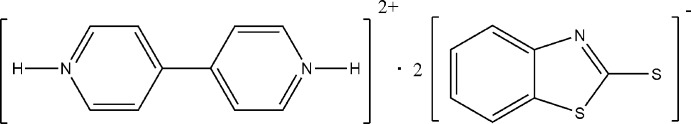


## Experimental

### 

#### Crystal data

C_10_H_10_N_2_^2+^·2C_7_H_4_NS_2_^−^
*M* *_r_* = 490.66
Monoclinic, 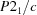

*a* = 14.3909 (13) Å
*b* = 5.6670 (4) Å
*c* = 15.5471 (14) Å
β = 109.023 (2)°
*V* = 1198.67 (17) Å^3^
*Z* = 2
Mo *K*α radiation
μ = 0.42 mm^−1^
*T* = 298 K
0.32 × 0.30 × 0.26 mm

#### Data collection

Bruker SMART CCD area-detector diffractometer
Absorption correction: multi-scan (*SADABS*; Bruker, 2007 ▶) *T*_min_ = 0.878, *T*_max_ = 0.900
5663 measured reflections
2116 independent reflections
990 reflections with *I* > 2σ(*I*)
*R*_int_ = 0.055

#### Refinement

*R*[*F* ^2^ > 2σ(*F* ^2^)] = 0.060
*wR*(*F* ^2^) = 0.203
*S* = 1.04
2116 reflections
145 parameters
H-atom parameters constrained
Δρ_max_ = 0.29 e Å^−3^
Δρ_min_ = −0.26 e Å^−3^

### 

Data collection: *SMART* (Bruker, 2007 ▶); cell refinement: *SAINT-Plus* (Bruker, 2007 ▶); data reduction: *SAINT-Plus*; program(s) used to solve structure: *SHELXS97* (Sheldrick, 2008 ▶); program(s) used to refine structure: *SHELXL97* (Sheldrick, 2008 ▶); molecular graphics: *SHELXTL* (Sheldrick, 2008 ▶); software used to prepare material for publication: *SHELXTL*.

## Supplementary Material

Crystal structure: contains datablock(s) global, I. DOI: 10.1107/S1600536812047058/jj2151sup1.cif

Structure factors: contains datablock(s) I. DOI: 10.1107/S1600536812047058/jj2151Isup2.hkl

Additional supplementary materials:  crystallographic information; 3D view; checkCIF report

## Figures and Tables

**Table 1:** Hydrogen-bond geometry (Å, °)

*D*—H⋯*A*	*D*—H	H⋯*A*	*D*⋯*A*	*D*—H⋯*A*
N2—H2⋯N1^i^	0.86	1.93	2.790 (6)	178

Symmetry code: (i) 
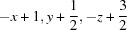
.

**Fig. 1.**
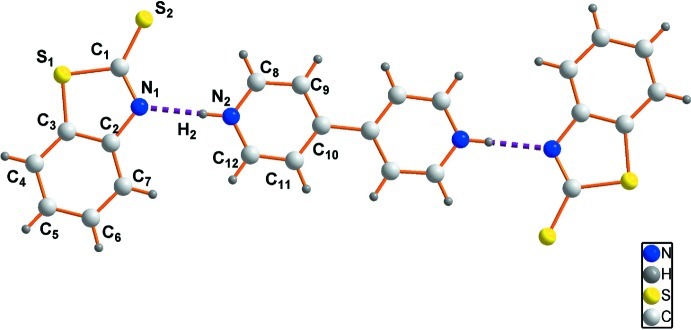
The molecular entities of the title compound, showing the atom-numbering scheme of the 4-pyridyl and mercaptobenzothiazole units and the symmetry expanded 4,4'-bipyridine-1,1'-diium cation and two mbt cation units with displacement ellipsoids drawn at the 50% probability level.

**Fig. 2.**
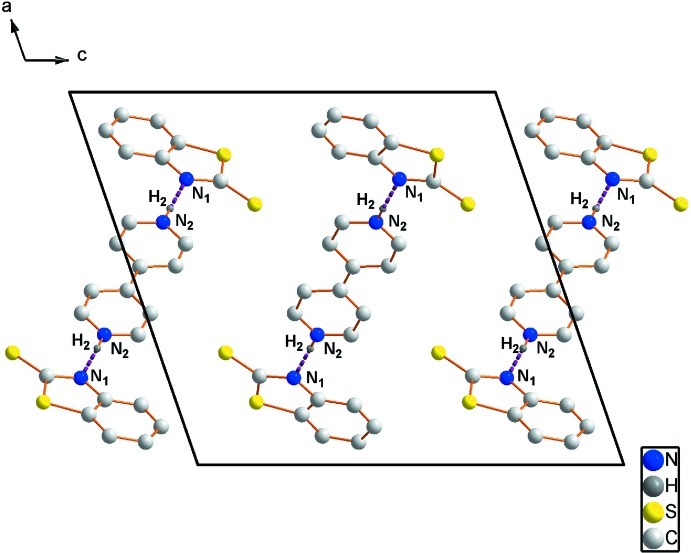
A view of the packing in (I) along the b axis. Dashed lines indicate N—H···N hydrogen bonds. H atoms not involved in hydrogen bonding have been removed for clarity.

## supplementary crystallographic information

### Comment

The 4,4'-bipyridine lignad is ideal for forming supramolecular structures. Many examples in coordination with multifarious metals are observed (Biradha *et al.*, 1999; Tong *et al.*, 2000; Tao *et al.*, 2000; Ren *et al.*, 2004; Xu *et al.*, 2012). However, to our best knowledge, only a few Ag(I)-Hmbt (Hmbt = 2-mercaptobenzothiazole) framework structures have been reported (Chen *et al.*, 2010). In our work synthesizing an Ag(I)-Hmbt complex containing the 4,4'-bipyridine, the title compound, (I), (C_10_H_10_N_2_).(C_7_H_4_NS_2_)_2_ was unexpectedly obtained.

The crystal structure of the title compound, (I), consists of one mbt (mercaptobenzothiazole) anion and one 4-pyridyl unit containg a center of symmetry which upon expansion produces a 4,4'-bipyridine-1,1'-diium cation and two mbt cations in the asymmetric unit (Fig. 1). Crystal packing reveals that N—H···N intermolecular hydrogen bonds are observed between the centrosymmetric 4,4'-bipyridine-1,1'-diium cation and two mbt anions (Fig. 2; Table 1). These observed hydrogen bonds are similar to those reported in a similar and related compound, (C_10_H_8_N_2_)(C_2_H_3_N_3_S_2_)_2_, (Deng *et al.*, 2005).

### Experimental

A mixture of AgBr (0.2 mmol) and 2-mercaptobenzothiazole (0.2 mmol) in MeOH and CH_2_Cl_2_ (10 mL, *v*/*v* = 1:1) was stirred for 2 h and triphenylphosphine (PPh_3_)(0.2 mmol) was added to the mixture which was stirred for another 5 h. The insoluble residues were removed by filtration. The filtrate was then evaporated slowly at room temperature for a week to yield colorless crystalline products. Anal. Calc. for C_24_H_18_N_4_S_4_: C, 58.70; H, 3.67; N, 11.41. Found: C, 58.49; H, 3.79; N, 11.22%. Melting point: 427–431°K.

### Refinement

All H atoms were located in the calculated sites and included in the final refinement in the riding model approximation with displacement parameters derived from the parent atoms to which they were bonded (*U*_iso_(H) = 1.2Ueq). C—H hydrogen atoms (aromatic) were included with distance set to 0.93 Å and amide N—H hydrogen atoms were included with distance set to 0.86 Å.

### Crystal data

**Table:**

C_10_H_10_N_2_^2+^·2C_7_H_4_NS_2_^−^	*F*(000) = 508
*M**_r_* = 490.66	*D*_x_ = 1.359 Mg m^−^^3^
Monoclinic, *P*2_1_/*c*	Mo *K*α radiation, λ = 0.71073 Å
Hall symbol: -P 2ybc	Cell parameters from 1198 reflections
*a* = 14.3909 (13) Å	θ = 2.7–20.7°
*b* = 5.6670 (4) Å	µ = 0.42 mm^−^^1^
*c* = 15.5471 (14) Å	*T* = 298 K
β = 109.023 (2)°	Block, colorless
*V* = 1198.67 (17) Å^3^	0.32 × 0.30 × 0.26 mm
*Z* = 2	

### Data collection

**Table:**

Bruker SMART CCD area-detector diffractometer	2116 independent reflections
Radiation source: fine-focus sealed tube	990 reflections with *I* > 2σ(*I*)
Graphite monochromator	*R*_int_ = 0.055
phi and ω scans	θ_max_ = 25.0°, θ_min_ = 2.7°
Absorption correction: multi-scan (*SADABS*; Bruker, 2007)	*h* = −17→11
*T*_min_ = 0.878, *T*_max_ = 0.900	*k* = −6→6
5663 measured reflections	*l* = −18→18

### Refinement

**Table:**

Refinement on *F*^2^	Primary atom site location: structure-invariant direct methods
Least-squares matrix: full	Secondary atom site location: difference Fourier map
*R*[*F*^2^ > 2σ(*F*^2^)] = 0.060	Hydrogen site location: inferred from neighbouring sites
*wR*(*F*^2^) = 0.203	H-atom parameters constrained
*S* = 1.04	*w* = 1/[σ^2^(*F*_o_^2^) + (0.0797*P*)^2^ + 0.6601*P*] where *P* = (*F*_o_^2^ + 2*F*_c_^2^)/3
2116 reflections	(Δ/σ)_max_ < 0.001
145 parameters	Δρ_max_ = 0.29 e Å^−^^3^
0 restraints	Δρ_min_ = −0.26 e Å^−^^3^

### Special details

**Table:**

Geometry. All e.s.d.'s (except the e.s.d. in the dihedral angle between two l.s. planes) are estimated using the full covariance matrix. The cell e.s.d.'s are taken into account individually in the estimation of e.s.d.'s in distances, angles and torsion angles; correlations between e.s.d.'s in cell parameters are only used when they are defined by crystal symmetry. An approximate (isotropic) treatment of cell e.s.d.'s is used for estimating e.s.d.'s involving l.s. planes.
Refinement. Refinement of *F*^2^ against ALL reflections. The weighted *R*-factor *wR* and goodness of fit *S* are based on *F*^2^, conventional *R*-factors *R* are based on *F*, with *F* set to zero for negative *F*^2^. The threshold expression of *F*^2^ > σ(*F*^2^) is used only for calculating *R*-factors(gt) *etc*. and is not relevant to the choice of reflections for refinement. *R*-factors based on *F*^2^ are statistically about twice as large as those based on *F*, and *R*- factors based on ALL data will be even larger.

### Fractional atomic coordinates and isotropic or equivalent isotropic displacement parameters (Å^2^)

**Table:**

	*x*	*y*	*z*	*U*_iso_*/*U*_eq_	
N1	0.2335 (3)	0.3414 (7)	0.7956 (2)	0.0629 (11)	
N2	0.6509 (3)	0.4638 (7)	0.6145 (3)	0.0700 (11)	
H2	0.6875	0.5792	0.6419	0.084*	
S1	0.16856 (12)	0.6851 (3)	0.68804 (10)	0.1018 (7)	
S2	0.30080 (16)	0.3128 (4)	0.65559 (11)	0.1352 (9)	
C1	0.2387 (4)	0.4264 (10)	0.7171 (3)	0.0812 (16)	
C2	0.1755 (3)	0.4737 (9)	0.8346 (3)	0.0586 (12)	
C3	0.1353 (4)	0.6727 (10)	0.7852 (3)	0.0720 (14)	
C4	0.0782 (4)	0.8244 (11)	0.8160 (5)	0.102 (2)	
H4	0.0515	0.9598	0.7835	0.122*	
C5	0.0617 (5)	0.7703 (13)	0.8960 (6)	0.112 (2)	
H5	0.0217	0.8680	0.9170	0.135*	
C6	0.1030 (5)	0.5758 (13)	0.9448 (4)	0.0991 (19)	
H6	0.0921	0.5448	0.9995	0.119*	
C7	0.1601 (3)	0.4255 (9)	0.9152 (3)	0.0700 (14)	
H7	0.1879	0.2929	0.9491	0.084*	
C8	0.5934 (4)	0.3548 (11)	0.6502 (4)	0.0888 (18)	
H8	0.5926	0.4038	0.7070	0.107*	
C9	0.5343 (4)	0.1726 (11)	0.6091 (4)	0.0888 (17)	
H9	0.4955	0.0979	0.6385	0.107*	
C10	0.5317 (3)	0.0987 (8)	0.5244 (3)	0.0549 (12)	
C11	0.5936 (4)	0.2124 (10)	0.4888 (3)	0.0843 (17)	
H11	0.5967	0.1676	0.4322	0.101*	
C12	0.6507 (4)	0.3907 (11)	0.5350 (4)	0.0914 (19)	
H12	0.6923	0.4653	0.5086	0.110*	

### Atomic displacement parameters (Å^2^)

**Table:**

	*U*^11^	*U*^22^	*U*^33^	*U*^12^	*U*^13^	*U*^23^
N1	0.068 (2)	0.077 (3)	0.046 (2)	−0.008 (2)	0.0210 (19)	0.002 (2)
N2	0.060 (2)	0.070 (3)	0.072 (3)	−0.015 (2)	0.010 (2)	−0.002 (2)
S1	0.1082 (12)	0.1031 (13)	0.0740 (9)	−0.0164 (10)	0.0019 (8)	0.0351 (9)
S2	0.1740 (19)	0.177 (2)	0.0824 (11)	−0.0039 (16)	0.0806 (12)	0.0087 (12)
C1	0.086 (4)	0.105 (5)	0.051 (3)	−0.020 (3)	0.020 (3)	0.013 (3)
C2	0.057 (3)	0.059 (3)	0.054 (3)	−0.004 (2)	0.010 (2)	0.001 (2)
C3	0.063 (3)	0.058 (3)	0.077 (3)	−0.009 (3)	−0.002 (3)	0.005 (3)
C4	0.074 (4)	0.061 (4)	0.141 (6)	0.008 (3)	−0.004 (4)	0.000 (4)
C5	0.091 (5)	0.094 (6)	0.146 (7)	0.011 (4)	0.031 (5)	−0.035 (5)
C6	0.103 (5)	0.105 (5)	0.095 (4)	0.012 (4)	0.040 (4)	−0.018 (4)
C7	0.076 (3)	0.069 (3)	0.068 (3)	0.005 (3)	0.027 (3)	−0.004 (3)
C8	0.089 (4)	0.108 (5)	0.077 (4)	−0.031 (4)	0.038 (3)	−0.019 (3)
C9	0.085 (4)	0.116 (5)	0.081 (4)	−0.027 (4)	0.049 (3)	−0.015 (4)
C10	0.044 (3)	0.061 (3)	0.057 (3)	0.004 (2)	0.013 (2)	0.011 (2)
C11	0.099 (4)	0.103 (4)	0.054 (3)	−0.035 (4)	0.029 (3)	−0.007 (3)
C12	0.109 (4)	0.112 (5)	0.058 (3)	−0.040 (4)	0.033 (3)	0.001 (3)

### Geometric parameters (Å, º)

**Table:**

N1—C1	1.337 (5)	C5—H5	0.9300
N1—C2	1.399 (5)	C6—C7	1.363 (7)
N2—C8	1.294 (6)	C6—H6	0.9300
N2—C12	1.303 (6)	C7—H7	0.9300
N2—H2	0.8600	C8—C9	1.358 (7)
S1—C3	1.728 (6)	C8—H8	0.9300
S1—C1	1.754 (6)	C9—C10	1.371 (6)
S2—C1	1.638 (6)	C9—H9	0.9300
C2—C7	1.370 (6)	C10—C11	1.355 (6)
C2—C3	1.381 (6)	C10—C10^i^	1.487 (8)
C3—C4	1.378 (8)	C11—C12	1.352 (7)
C4—C5	1.375 (9)	C11—H11	0.9300
C4—H4	0.9300	C12—H12	0.9300
C5—C6	1.361 (9)		
			
C1—N1—C2	115.0 (4)	C5—C6—H6	119.3
C8—N2—C12	116.8 (5)	C7—C6—H6	119.3
C8—N2—H2	121.6	C6—C7—C2	118.6 (5)
C12—N2—H2	121.6	C6—C7—H7	120.7
C3—S1—C1	92.4 (3)	C2—C7—H7	120.7
N1—C1—S2	126.5 (5)	N2—C8—C9	123.5 (5)
N1—C1—S1	109.7 (4)	N2—C8—H8	118.3
S2—C1—S1	123.8 (3)	C9—C8—H8	118.3
C7—C2—C3	120.6 (5)	C8—C9—C10	120.1 (5)
C7—C2—N1	126.0 (4)	C8—C9—H9	120.0
C3—C2—N1	113.4 (4)	C10—C9—H9	120.0
C4—C3—C2	120.3 (5)	C11—C10—C9	115.6 (4)
C4—C3—S1	130.1 (5)	C11—C10—C10^i^	121.7 (5)
C2—C3—S1	109.6 (4)	C9—C10—C10^i^	122.7 (5)
C5—C4—C3	118.3 (6)	C12—C11—C10	120.3 (5)
C5—C4—H4	120.9	C12—C11—H11	119.8
C3—C4—H4	120.9	C10—C11—H11	119.8
C6—C5—C4	120.8 (6)	N2—C12—C11	123.7 (5)
C6—C5—H5	119.6	N2—C12—H12	118.1
C4—C5—H5	119.6	C11—C12—H12	118.1
C5—C6—C7	121.3 (6)		
			
C2—N1—C1—S2	179.6 (4)	C3—C4—C5—C6	1.9 (10)
C2—N1—C1—S1	0.0 (5)	C4—C5—C6—C7	−1.6 (10)
C3—S1—C1—N1	0.9 (4)	C5—C6—C7—C2	0.1 (8)
C3—S1—C1—S2	−178.8 (4)	C3—C2—C7—C6	1.0 (7)
C1—N1—C2—C7	−178.8 (4)	N1—C2—C7—C6	178.6 (5)
C1—N1—C2—C3	−1.1 (6)	C12—N2—C8—C9	−0.2 (8)
C7—C2—C3—C4	−0.6 (7)	N2—C8—C9—C10	−1.5 (9)
N1—C2—C3—C4	−178.5 (4)	C8—C9—C10—C11	2.4 (8)
C7—C2—C3—S1	179.5 (4)	C8—C9—C10—C10^i^	−179.7 (5)
N1—C2—C3—S1	1.7 (5)	C9—C10—C11—C12	−1.8 (8)
C1—S1—C3—C4	178.7 (5)	C10^i^—C10—C11—C12	−179.7 (5)
C1—S1—C3—C2	−1.5 (4)	C8—N2—C12—C11	0.9 (8)
C2—C3—C4—C5	−0.8 (8)	C10—C11—C12—N2	0.1 (9)
S1—C3—C4—C5	179.0 (5)		

Symmetry code: (i) −*x*+1, −*y*, −*z*+1.

### Hydrogen-bond geometry (Å, º)

**Table:**

*D*—H···*A*	*D*—H	H···*A*	*D*···*A*	*D*—H···*A*
N2—H2···N1^ii^	0.86	1.93	2.790 (6)	178

Symmetry code: (ii) −*x*+1, *y*+1/2, −*z*+3/2.
